# In Toto Adipocytes Analysis Using Hydrophilic Tissue Clearing, Light Sheet Microscopy, and Deep Learning‐Based Image Processing

**DOI:** 10.1111/boc.70013

**Published:** 2025-06-22

**Authors:** Dylan Le Jan, Manar Harb, Mohamed Siliman Misha, Jean‐Claude Desfontis, Yassine Mallem, Laurence Dubreil

**Affiliations:** ^1^ Oniris, NP3 (Nutrition, PathoPhysiology & Pharmacology) Nantes France; ^2^ Oniris INRAE APEX PAnTher Nantes France

**Keywords:** adipocyte volume, image analysis, light sheet microscopy, mesenteric adipose tissue, tissue clearing

## Abstract

**Background Information:**

Obesity is a multifactorial metabolic disease characterized by excessive fat storage in adipocytes, particularly in visceral adipose tissue (VAT) like mesenteric adipocytes. Metabolic dysfunctions due to obesity are often associated with modification of adipocyte volume. Various techniques for measuring adipocyte size are described in the literature, including classical histological methods on paraffin‐embedded tissue sections or dissociation of adipose tissue (AT) using collagenase with artifacts due to AT post treatment.

**Results:**

This study aims to develop and implement an innovative method for 3D investigation of AT to assess adipocyte volume, overcoming the limitations and biases inherent in traditional techniques. The principle of the method relies on fluorescent labeling of lipids and extracellular matrix (ECM) in toto within AT, followed by a tissue clearing step without delipidation and imaging using 3D light sheet microscopy coupled with automated analysis of adipocyte size through a deep learning approach. By this work we showed that the volume of adipocytes increased in mesenteric AT from obese rats with an increase in the distance between adipocytes.

**Conclusion and Significance:**

The current work highlights the interest in combining AT clearing without a delipidation step and light sheet microscopy for in toto 3D adipocyte characterization in obese versus healthy rats. While this method is particularly valuable for understanding adipocyte hypertrophy in the context of obesity, its applicability extends beyond this area. This innovative approach offers valuable opportunities for investigating adipocyte dynamics in various pathological conditions, evaluating the impact of nutritional interventions, and assessing the effectiveness of pharmacological treatments.

## Graphical abstract

This manuscript presents a workflow for the 3D analysis of adipose tissue. The method is based on fluorescent labeling of lipids and extracellular matrix in toto within adipose tissue, followed by a tissue clearing and imaging using 3D LightSheet microscopy. Automated analysis of adipocyte size is then performes using a deep learning approach. This methode enables the structural characterization of adipose tissue architecture by measuring morphological parameters.

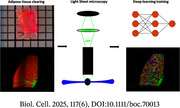

**Abbreviations**
5‐DTAF: 5‐(4,6‐dichlorotriazinyl)aminofluorescein
AT: adipose tissue
ECM: extracellular matrix
GA3: general analysis 3
HFD: high‐fat diet
NOD: nearest object distance
RI: refractive index
VAT: visceral adipose tissue

## 1 Introduction

Obesity is a complex metabolic disorder influenced by various factors, including environmental conditions, diet, and genetics (Hruby and Hu 2015). The central mechanism underlying obesity is an imbalance between caloric intake and energy expenditure, leading to excess fat storage, primarily in adipocytes located in visceral adipose tissue (VAT), such as mesenteric adipocytes (Ali et al. 2013; Frayn 1993). Hypertrophy of these adipocytes, often accompanied by hypoxia, leads to systemic inflammation and insulin resistance, both of which are key contributors to obesity‐related metabolic complications (Chan and Hsieh 2017).

The evaluation of adipocyte size is a crucial indicator of structural changes associated with obesity (Theobalt et al. 2021). An increased adipocyte volume has been identified as a predictive factor for the higher risk of obesity‐related type 2 diabetes, with specific volume thresholds correlating with greater insulin resistance (Cotillard et al. 2014). Several techniques for measuring adipocyte size are described in the literature, including classic histological methods on paraffin‐embedded tissue sections, dissociation of adipose tissue (AT) using collagenase to measure the size of isolated adipocytes (Björnheden et al. 2004), and flow cytometry, which also represents a useful tool for evaluating adipocyte volume (Hagberg et al. 2018).

2D approaches, which focus on parameters like cell diameter or surface area, although useful for basic analyses, do not provide reliable measurements of adipocyte volume (Laforest et al. 2015). Indeed, traditional 2D methods are prone to operator‐dependent errors and require labor‐intensive manual analysis (Chen and Farese 2002). This is illustrated by 2D studies showing that the equivalent diameter index can reveal up to 23% uncertainty in glioma size due to variations in scanning conditions (Schmitt et al. 2013). Furthermore, the analysis of adipocyte size on thin histological paraffin sections requires a tissue delipidation step, which can alter adipocyte structure and prevent access to cellular volume. Additionally, dissociating tissue with collagenase allows adipocytes to be freed from the tissue but also releases stromal/vascular cells that can contaminate the samples and bias the results (Majka et al. 2014). This limitation is particularly relevant in flow cytometry, where the presence of non‐adipocyte cells can interfere with gating strategies and affect the accuracy of adipocyte volume estimation. Moreover, the large size and fragility of adipocytes make them prone to deformation or rupture during flow‐based measurements, further limiting the reliability of this approach. Furthermore, 3D imaging of adipocytes is limited to a few tens of micrometers in confocal microscopy (Blackshear et al. 2018) due to the opacity of AT, which causes light scattering. Indeed, the heterogeneous composition of tissues (lipids, carbohydrates, proteins, and colored pigments bathed in interstitial fluids) results in a variety of refractive indices (RIs) responsible for light scattering (Azaripour et al. 2016).

The tissue clearing technique, making tissues optically transparent, offers a promising solution and has become a powerful tool for in toto three‐dimensional analysis (Costa et al. 2019; Costantini et al. 2019). However, these techniques are still underused in obesity research, particularly when it is important to preserve lipid storage. Indeed, current tissue clearing techniques used on AT rely on organic solvents to remove storage lipids in order to study 3D structures of the tissue, such as vascularization, innervation, and crownlike structures (Chi et al. 2018; Geng et al. 2021), but they do not allow the analysis of adipocytes that are altered after delipidation steps.

In this study, we propose a new 3D approach to investigate preserved whole adipocytes in cleared AT. This new approach involves fluorescent staining of AT with Nile Red and 5‐(4,6‐dichlorotriazinyl)aminofluorescein (5‐DTAF) fluorescent probes for lipids and extracellular matrix (ECM), respectively. Then, fluorescent AT is cleared by immersion in a high RI solution (RapiClear) without a delipidation step and imaged by light sheet microscopy to be analyzed by developing a workflow combining AI segmentation and deep learning tools. These techniques enable an accurate evaluation of new critical parameters on adipocyte morphology, such as volume, density, and sphericity, for a more precise and comprehensive evaluation of structural changes in adipocytes associated with obesity. The consideration of these new morphologic parameters could contribute to understanding the processes of adipocyte hypertrophy, especially in the context of obesity, and could be used to assess therapies developed to manage obesity.

## 2 Materials and Methods

### 2.1 Animals and Tissue Sample Preparation

Six male Wistar rats (8 weeks old) were obtained from Janvier Labs (Le Genest‐Saint‐Isle, France) and acclimatized for 1 week prior to the study. The animals were housed in accordance with European standard ETS 123, under a controlled 12‐h light/dark cycle, with a temperature maintained at 22 ± 2°C and 50% relative humidity. This study received approval from the Ethics Committee on Animal Experimentation of the Pays de la Loire Region (APAFIS#33784‐2021112413036973v3, 13 December 2021), in compliance with the guidelines of the French National Research Council for the Care and Use of Laboratory Animals.

The rats were randomly divided into two groups (*n* = 3 per group). The healthy group was fed a standard diet (3430 Kliba Nafag, Kaiseraugst, Switzerland), while the obese group received a high‐fat diet (HFD). The HFD consisted of pellets (D12451 Research Diets, Lynge, Denmark) and sweetened condensed milk (Nestlé, Nantes, France), both provided ad libitum for 26 weeks. To ensure the proper induction of obesity through dietary intervention, no significant differences in body weight were observed between groups at the start of the study. After 26 weeks of either standard diet or HF diet, the healthy, and obese groups exhibited mean body weights of 623.00 ± 20.03 g and 735.27 ± 12.04 g, respectively, representing an approximate difference of 18.04%. In rats, moderate obesity is typically defined as a body weight increase ranging from 10% to 25% (Bagnol et al. 2012), allowing us to categorize the HFD‐fed animals as obese.

At the end of the study, the rats were euthanized by an overdose of intraperitoneal anesthetic (pentobarbital, Euthasol, Fort Worth, TX, USA), followed immediately by the collection of mesenteric AT.

### 2.2 AT Staining and Clearing

**Figure 1:**
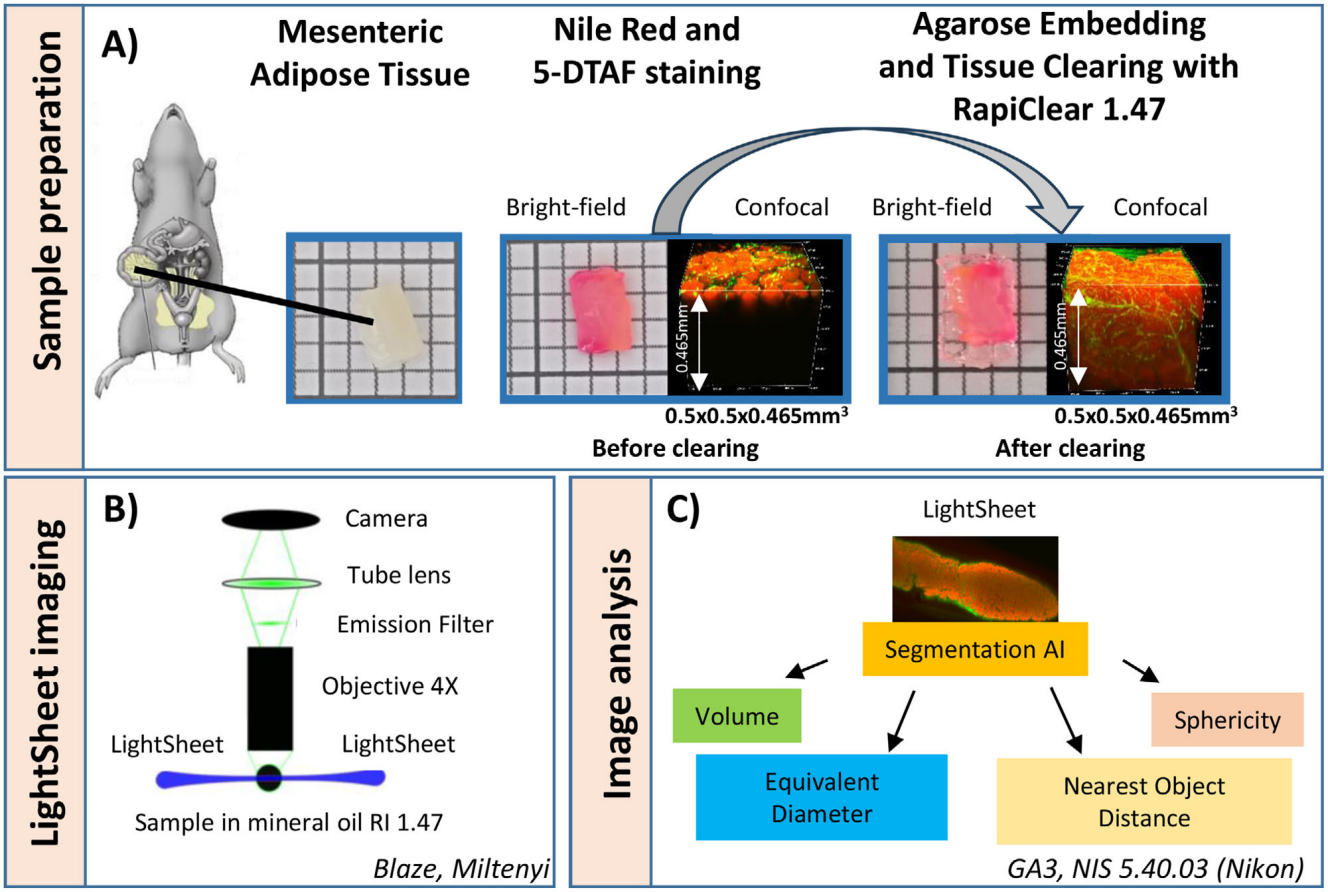
Methodological workflow for 3D imaging and analysis of mesenteric adipose tissue. (A) Sample preparation: Tissue is extracted from rat mesenteric adipose depots, stained with Nile Red and 5‐DTAF, embedded in agarose, and cleared using RapiClear 1.47. Bright‐field image and 3D confocal fluorescence images (0.5 × 0.5 × 0.465 mm^3^) of adipose tissue acquired before and after clearing. (B) Light sheet imaging: Cleared samples are imaged using a light sheet fluorescence microscope (Blaze, Miltenyi) with a 4× objective and mineral oil immersion (RI 1.47). (C) Image analysis: Segmentation AI processes the acquired light sheet images to extract key adipocyte morphological parameters, including volume, equivalent diameter, nearest object distance, and sphericity, using GA3 and NIS 5.40.03 software (Nikon).

Mesenteric AT was collected from rats after euthanasia. For fixing, samples were stored in 4% formaldehyde for at least 72 h. After cutting off part of the AT (around 3 mm thick), they were washed in PBS (Sigma; 806552) 3 times, 1 h each, with stirring (750 rpm) at room temperature. Stock solutions of Nile Red (Thermo Fisher, N1142‐25 mg, Waltham, MA, USA) and 5‐DTAF (Thermo Fisher, 11550216, Waltham, MA, USA) were prepared at 1 mg/mL in DMSO and at 10 mg/mL in PBS, respectively, and stored in the freezer at −20°C. A mixture of Nile Red and 5‐DTAF was diluted at 1/50 and 1/1000 in PBS, respectively. Samples were incubated first for 24 h in Nile Red 1/50. After that, the staining medium was replaced with mixture of Nile Red + 5‐DTAF and left to shake at room temperature for 24 h. Rinsing was carried out in PBS 3 times for 2 h each. To facilitate the handling and imaging of AT with the light sheet, samples were embedded in 2% agarose (low melting A0701, Sigma Aldrich, St. Louis, MO, USA) prior to clearing. Agarose was dissolved in PBS and heated using a water bath of 60°C to obtain a liquid solution. Samples were totally covered to create a mold. Samples were then removed from the mold after solidification, and agarose was trimmed as close as possible to the sample before immersion in RapiClear 1.47 (RapiClear, RC147001, Sun Jin Lab, Hsinchu City, Taiwan) for 2 days on a stirrer at room temperature (Figure 1). After that, RapiClear solution was changed and incubated for at least 24 h before imaging with the control of RI value by using a refractometer (Mettler Toledo, Portable Lab, France). This step was done to prevent the modification of RI by water releasing from agarose.

### 2.3 Light Sheet Imaging

Cleared samples were imaged with a Blaze light sheet microscope (Miltenyi Biotec, Bergisch Gladbach, Germany) equipped with an sCMOS 5.5 MP camera controlled by Inspector Pro acquisition software (Miltenyi Biotec, Bergisch Gladbach, Germany). Images were acquired with two different lasers: the 488 nm laser was used to excite the 5‐DTAF dye (for ECM staining), and the 561 nm laser was used for the excitation of the Nile Red dye (for lipid staining). The imaging was done with a 4× objective lens (NA 0.35). Samples were supported by a sample holder from Miltenyi and placed in a tank filled with mineral oil with RI 1.47 (M3516, Sigma Aldrich, St. Louis, MO, USA) and illuminated by the laser light sheet from both sides. light sheet thickness 4 µm, step size 4 µm. The images were acquired with a camera sCMOS, exposure time 50 ms, 2048 × 2048, pixel size 1.62 µm.

### 2.4 3D Images Analyses

**Figure 2:**
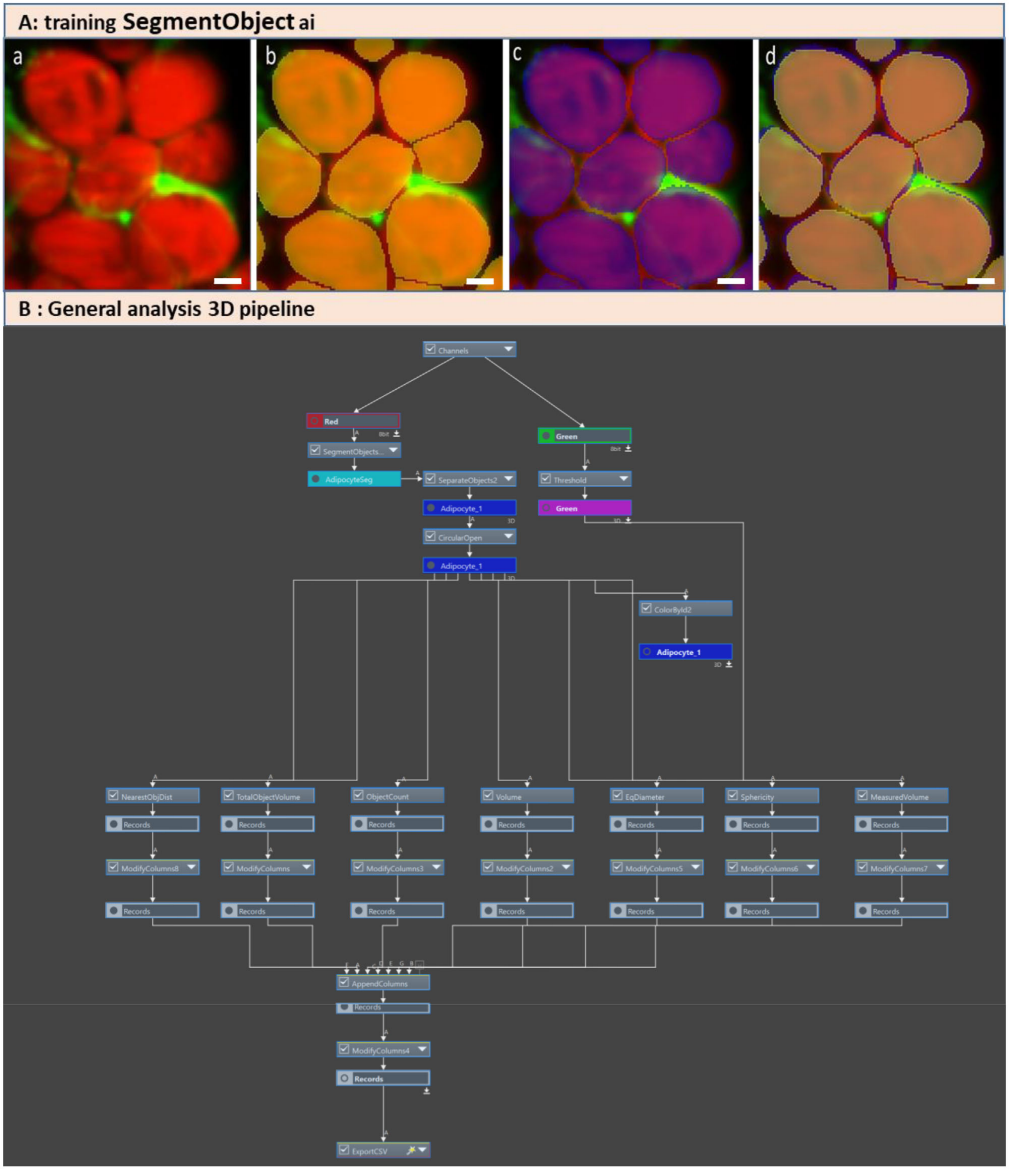
Deep learning‐based adipocyte segmentation and 3D image analysis pipeline. (A) Application of the algorithm SegmentObject.ai generated from training, the cropped images highlight the efficiency of the predictions generated by the deep learning models for adipocyte segmentation: **a**: Cropped image with adipocyte labeled with Nile Red (red) and extracellular matrix labeled with 5‐DTAF (green). **b**: Cropped image annotated using the initial version of the algorithm, followed by manual correction. **c**: Cropped image processed with the updated algorithm after training the deep learning model. **d**: Overlay of the segmentations produced by the initial and updated algorithms, demonstrating their alignment and differences. Scale bar = 25 µm. Learning model was also evaluated from Loss curve (training Loss 0.00279 with 1500 iterations). (B) General Analysis 3 pipeline (NIS‐Elements software version 5.42.03, Nikon Europe B.V.) for 3D image analyses including resulting segmentobject.ai algorithm to measure total object volume, object count, individual object volume, equivalent diameter, sphericity, and nearest object distance (NOD).

Light sheet images from cleared AT were post‐processed with Miltenyi software MACS to do a 3.32 mm × 3.32 mm × 0.4 mm crop and processed using NIS software Nikon 5.42.03 (Nikon Europe B.V., Amstelveen, The Netherlands). Due to variations in fluorescent signal intensity and the proximity of contiguous objects of interest, manual segmentation was deemed unsuitable as it introduced variability in the results. Consequently, we opted to implement a deep learning‐based segmentation approach to enhance the reproducibility and efficiency of the measurements (Figure 2). Segmentation was conducted using the U‐Net‐based deep learning model “SegmentObject.ai,” integrated into the NIS‐Elements software.

Deep learning training: The training algorithm was conducted in two rounds. In the first round, the dataset consisted of three images obtained from an averaged Z‐stack, with five frames per stack. The three representative planes—the first, middle, and last—were saved separately, followed by manual thresholding of the structure of interest. AT was initially identified using intensity‐based thresholding, with subsequent manual refinement to generate precise binary masks. These refined masks served as the ground truth for model training, which was carried out for up to 200 iterations.

The resulting algorithm was applied to a dataset comprising 16 images extracted using the same procedure. The binary mask images generated were manually corrected and cropped to produce final images containing between 5 and 10 adipocytes. These corrected binary masks from the 16 cropped images were used as the ground truth for the second round of training, which was conducted over 1500 iterations. This process resulted in a more robust and accurate segmentation algorithm (score training curve loss < 0.003). To verify its consistency with the previous version, the updated algorithm was applied to a cropped image as a validation step (Figure 2A).

The resulting algorithm was subsequently imported into a General Analysis 3 (GA3) pipeline for further feature measurements (Figure 2B). 3D images (2042.82 µm × 1885.68 µm × 380 µm) from 3 healthy and 3 obese rats were analyzed to assess key morphological features, including total object volume, object count, individual object volume, equivalent diameter, sphericity, and nearest object distance (NOD). The GA3 pipeline was developed to extract and quantify various features of AT from 3D images. This approach leverages deep learning‐based segmentation and advanced image processing techniques to investigate the structural organization of AT. The pipeline begins with the application of U‐Net‐based deep learning models. Segment.Object.ai is used for segmenting red‐stained adipocytes. A binary operation, “separate objects,” is applied to divide connected adipocyte structures into smaller individual objects. A second binary operation, “circular open,” is used to remove regions that cannot accommodate a circle or sphere of the adipocyte. Measurements are then performed, including: The number and volume of adipocytes, NOD, equivalent diameter and sphericity of the adipocyte, and the total object volume of both adipocytes and the ECM. This 3D pipeline provides an integrated framework for characterizing the structural organization of AT in a 3D context. The extracted data were subsequently exported for statistical analysis (Figure 2).

Each individual sample consisted of 450 analyzed adipocytes (*n* = 1350 adipocytes per group). Four key parameters were assessed to evaluate the morphology of the adipocytes. The first parameter was the 3D volume of each adipocyte. The second parameter, the NOD, corresponded to the smallest distance to another adipocyte (measured between centers of gravity). The third parameter was the equivalent diameter, which was calculated by the software as the diameter of a sphere with the same volume as the measured adipocyte. Finally, the sphericity of each adipocyte was evaluated, which was computed as the ratio of the adipocyte surface and the surface of a sphere with matching volume. A sphere has the maximum sphericity = 1.

### 2.5 Statistical Analysis

Results are expressed as mean ± standard error of the mean (SEM). Statistical analyses were performed using GraphPad Prism (v.9.0.0). Comparisons of means were conducted using Mann–Whitney tests, while distribution analyses were performed using multiple Mann–Whitney tests for pairwise comparisons between groups, with multiple comparisons corrected using the false discovery rate approach at a *Q* value of 5%. A *p* value < 0.05 was considered statistically significant.

## 3 Results

### 3.1 3D Visualization of Adipocyte From Nile Red Staining, RapiClear Clearing of AT Combined With Light Sheet Microscopy

Lipids were stained with Nile Red, in accordance with published data (Greenspan et al. 1985), while the ECM was stained with 5‐DTAF (Graham et al. 2017). The 3D structure of mesenteric adipocytes within AT was investigated using RapiClear, a clearing reagent that preserves storage lipids (Chen et al. 2021; Horckmans et al. 2022) and retaining the fluorescent labeling (Figure 1). Unlike solvent‐based clearing techniques such as uDISCO (Pan et al. 2016), which typically cause tissue rigidification, AT cleared with RapiClear remained soft and flexible. To facilitate light sheet imaging, we embedded the tissue in agarose before the clearing process.

**Figure 3:**
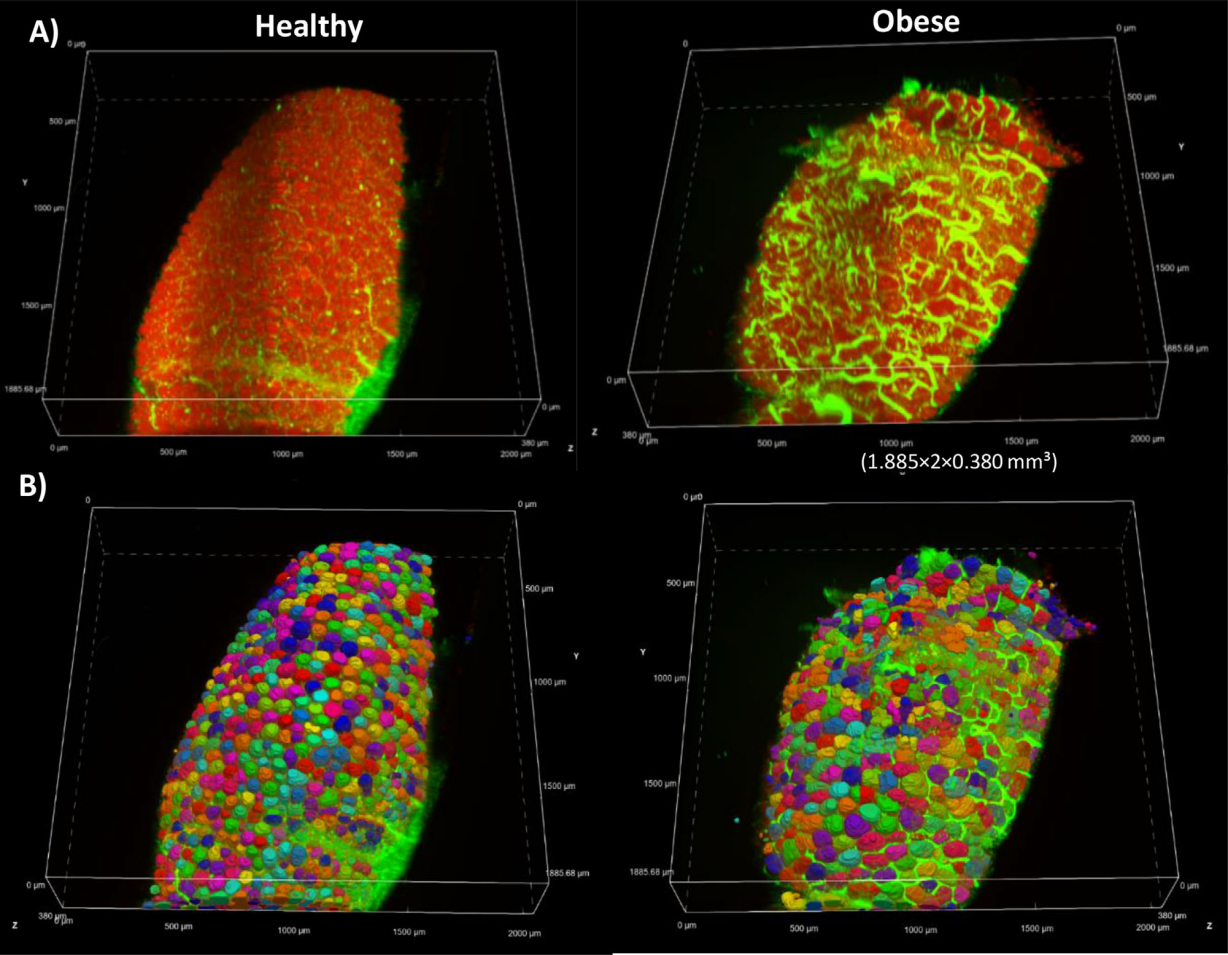
3D Visualization and Segmentation of Cleared Adipose Tissue in healthy and obese Groups. (A) 3D visualization of RapiClear 1.47 cleared adipose tissue stained with Nile Red (adipocytes in red) and 5‐DTAF (extracellular matrix in green) in healthy and obese groups. Observation with light sheet ultramicroscope Blaze, objective 4 × (1.885 × 2 × 0.380 mm^3^). (B) segmented 3D adipocytes using the SegmentObject.ai module in NIS‐Elements software (version 5.42.03, Nikon Europe BV, Amstelveen).

Images were then acquired using the Ultramicroscope Blaze, equipped with a 4× objective and an immersion tank filled with mineral oil (RI 1.467), closely matching the RI 1.47 of RapiClear (Figure 1). Adipocytes as well as the ECM surrounding the adipocytes in situ within AT was imaged over several millimeters in surface area but the depth was limited to half millimeter. Indeed, the clearing of AT was not fully optimized, as storage lipids significantly contribute to tissue opacity (Tian et al. 2021). A 3D reconstruction of RapiClear 1.47 cleared AT stained with Nile Red (adipocyte lipids in red) and 5‐DTAF (ECM in green) in both healthy and obese animal is shown in Figure 3.

Image quality was acceptable for depths up to 400 µm, which allowed for correct segmentation of objects using the segmentobject.ai algorithm developed from NIS. The segmentation process was refined through successive annotations and training cycles. The quality of the segmentation and consequently the deep learning algorithm, was validated by the loss training curve (score 0.00279; 1500 iterations) and overlay of the annotation with segmentation produced by updated algorithms (Figure 2). This analysis was performed on healthy and obese groups after 26 weeks on a diet to quantify morphological characteristics and distribution patterns of adipocytes into AT.

### 3.2 3D New Features to Characterize Adipocytes in Tissue: Volume, Density, Diameter, Sphericity

**Figure 4:**
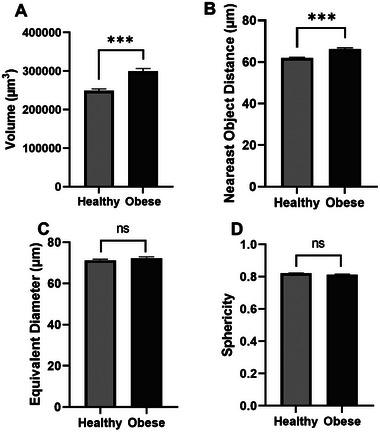
Evaluation of adipocyte characteristics in the healthy and obese groups: (A) adipocyte volume (µm^3^); (B) nearest object distance (NOD, µm) between adipocytes; (C) adipocyte equivalent diameter (µm); and (D) adipocyte sphericity. Quantification was done using a fully automated image processing algorithm using NIS‐Elements software. All results are expressed as mean ± standard error of the mean, with *n* = 1647 adipocytes. Statistical significance was determined using Mann–Whitney tests. ***: *p* value < 0.001; ns: non‐significant.

The mean adipocyte volume was measured on 450 adipocytes of each sample (Figure 4A). A Significant difference was observed between adipocyte volume of obese group (299,242 ± 7342 µm^3^) and the healthy group (248,754 ± 5129 µm^3^), reflecting an approximate 20.30% increase in volume with obesity (*p* < 0.001). In addition, the mean NOD measured between adipocytes was significantly higher in the obese group (66.23 ± 0.64 µm) compared to the healthy group (61.91 ± 0.43 µm), representing a 6.98% increase (*p* < 0.001) (Figure 4B).

In contrast, the mean equivalent diameter, did not show a statistically significant difference between the groups. The obese group had a mean equivalent diameter of 72.13 ± 0.76 µm, while the healthy group measured 71.17 ± 0.58 µm (Figure 4C). Similarly, mean sphericity remained relatively stable between conditions, with the obese group displaying a mean value of 0.813 ± 0.002 and the healthy group at 0.820 ± 0.002, indicating a difference of less than 1% (Figure 4D).

### 3.3 3D features Distribution Provides Additional Insights to Characterize AT

**Figure 5:**
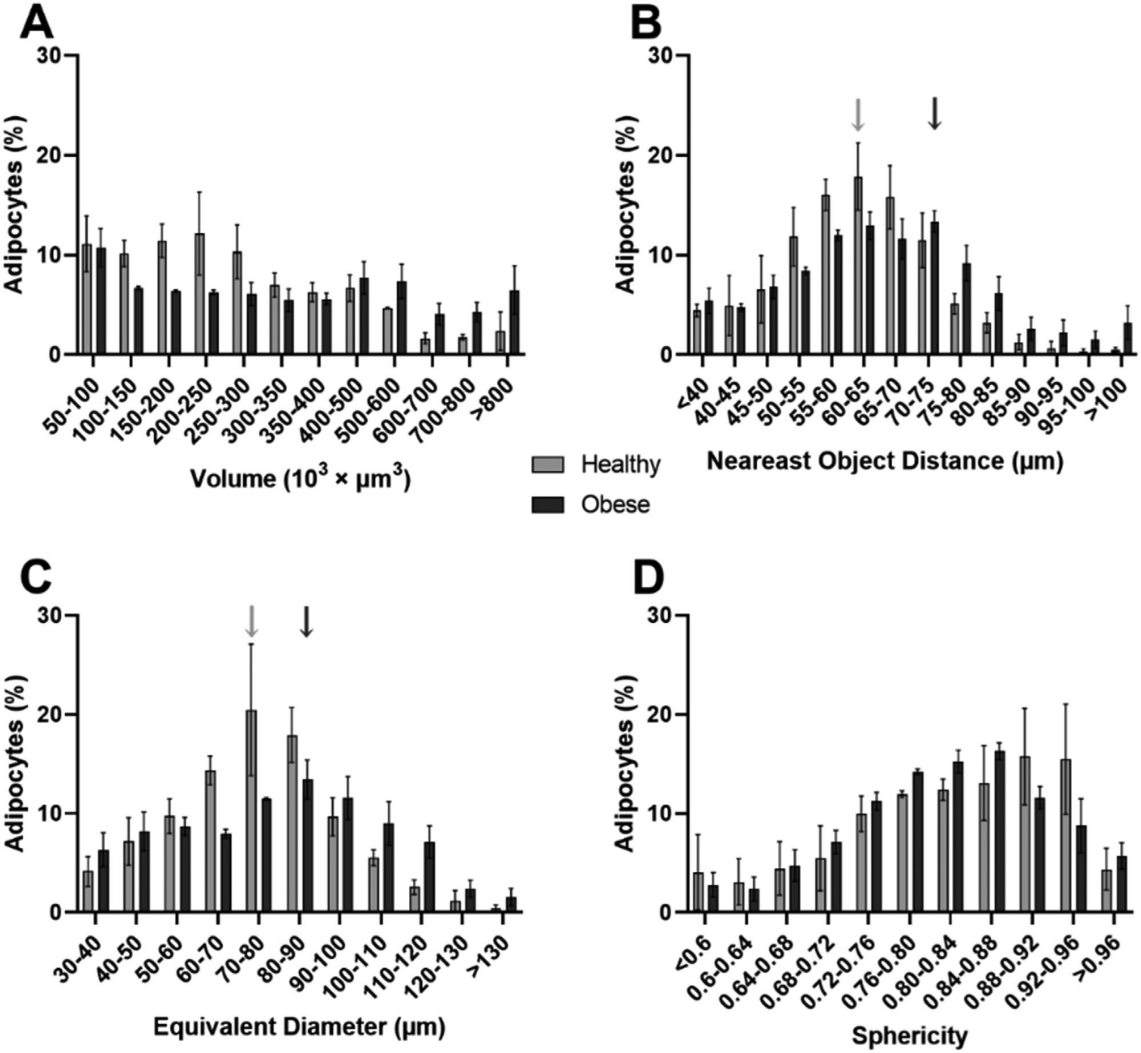
Distribution of adipocyte characteristics in the healthy and obese groups: (A) adipocyte volume (µm^3^), (B) nearest object distance (NOD, µm), (C) adipocyte equivalent diameter (µm), and (D) adipocyte sphericity. Arrows indicate the location of the maximal peak (light for healthy and dark for obese) for NOD and equivalent diameter. All results are expressed as the mean number of adipocytes in each class with *n* = 3 rats. Statistical significance was determined using multiple Mann–Whitney tests; however, no significant differences were observed between the groups.

The distribution of adipocyte volume exhibited distinct patterns between the two diet groups. In the healthy group, adipocytes were predominantly found in moderate volume categories (100,000–300,000 µm^3^), whereas the obese group contained a higher number of adipocytes in larger volume classes, particularly those exceeding 500,000 µm^3^ (Figure 5A).

Similarly, NOD distribution varied between groups. In the healthy group, adipocytes were most prevalent in medium‐distance categories (50–70 µm), while the obese group displayed a greater frequency in higher NOD values. A peak was observed in the 60–65 µm range for the healthy group, whereas the obese group peaked in the 70–75 µm range (Figure 5B).

For equivalent diameter, the obese group showed a larger proportion of adipocytes in higher diameter classes (>100 µm), while the healthy group had more adipocytes in moderate ranges (60–90 µm). The distribution peaked at 70–80 µm for the healthy group and 80–90 µm for the obese group (Figure 5C).

Finally, the distribution of sphericity appeared comparable between the two groups. The healthy group showed a slight predominance in higher sphericity categories, while the obese group had a greater frequency in medium sphericity ranges, particularly within the 0.76–0.88 range (Figure 5D).

## 4 Discussion

The aim of this study was to develop a method for the 3D analysis of adipocyte morphological characteristics in mesenteric AT from both obese and healthy rats while preserving adipocyte integrity. VAT was specifically chosen due to its central role in metabolic dysfunctions associated with obesity, particularly those induced by an obesogenic diet. In rats, different anatomical regions are prone to fat accumulation, including epididymal, perirenal, and mesenteric fat depots (Tchkonia et al. 2013).

Unlike subcutaneous AT, VAT is metabolically more active and plays a critical role in systemic inflammation regulation, insulin resistance, and lipid metabolism alteration (Hajer et al. 2008). VAT is also a key producer of adipokines and modulates the inflammatory environment through macrophage‐derived pro‐inflammatory cytokines, such as IL‐6 and TNF‐α, which are elevated in obesity and contribute to chronic low‐grade inflammation (Balistreri et al. 2010). Mesenteric AT, located around the intestines, is particularly relevant as it plays a crucial role in local and systemic inflammation, it directly impact metabolic diseases such as insulin resistance, and is tightly connected to the intestinal barrier and microbiota (Kredel and Siegmund 2014). These characteristics make it a critical site for evaluating the effects of an obesogenic diet on obesity progression.

3D exploration of AT through microscopy requires optical clearing techniques to reduce light scattering. A major challenge was clearing the tissue while preserving adipocyte structures, as most clearing techniques involve a delipidation step to remove the lipids mainly responsible of the tissue opacity. Our gentle clearing method, based on simple immersion in a high‐refractive‐index solution (RapiClear), successfully preserved lipids, minimized tissue deformation, and preserves fluorescence staining (Gomariz et al. 2018; Lallemant et al. 2020).

Many organic solvents used for tissue clearing cause a substantial dehydration and, thus, tissue shrinkage, as well as the quenching of fluorescent staining or fluorescent protein emission (Tian et al. 2021). Notably, RapiClear is compatible with preserving endogenous fluorescent proteins such as GFP and YFP (Aihara et al. 2020; Stefaniuk et al. 2016), enhancing the versatility of our method. These fluorescent proteins are widely used for studying metabolic disorders, inflammation in AT (Neugebauer et al. 2024), adipocyte progenitor differentiation (Girousse et al. 2019), and monitoring of the AT browning process (Chan et al. 2018), providing insights into metabolic adaptations and therapeutic strategies for obesity.

Using RapiClear immersion, AT imaging in our study was limited to 400 µm in depth. However, this limitation was offset by the ability to perform rapid imaging of large samples thanks to light sheet microscopy (Ryu et al. 2023). This approach is particularly suitable for studying large‐scale AT architecture while maintaining high‐resolution structural details. Furthermore, the cost‐effectiveness of mineral oil with a refractive index of 1.47, used as an imaging medium in the light sheet tank, offers a financially viable alternative for large‐scale or repeated studies.

Our findings align with existing literature, although 3D studies on adipocyte volume remain scarce. For instance, studies on mice fed a HFD for 11 weeks reported increased adipocyte surface area in gonadal AT (LeMieux et al. 2015). Similarly, Ramírez et al. (2017) observed a higher area and equivalent diameter in visceral adipocytes of rats subjected to an HFD for 8 weeks. Jing et al. (2021) reported that adipocytes in rats on a 6‐week HFD were engorged with fat, with significantly larger equivalent diameters, perimeters, and areas.

While these studies consistently highlight adipocyte size enlargement, our results indicate that adipocyte volume is a more relevant parameter than equivalent diameter in our model. Unlike 2D measurements, 3D imaging captures the full spatial dimensions of adipocytes, enabling more comprehensive characterization of AT. 3D analysis revealed NOD as a new discriminant parameter between obese and healthy animals with an increase of the distance between nearest adipocytes in obese samples. This NOD increase may indicate tissue disorganization and be linked to pathological ECM accumulation, particularly collagen, within obese AT (Ruiz‐Ojeda et al. 2019). As adipocytes enlarge, excessive ECM buildup may restrict adipocyte expansion, impair angiogenesis, and exacerbate local inflammation and metabolic dysfunctions (Sun et al. 2011). In the context of obesity, AT inflammation marked by macrophage accumulation around dying adipocytes in “crown‐like structures” disrupts tissue architecture. Macrophage infiltration and adipocyte necrosis compromise cellular integrity, potentially explaining the observed increase in NOD. These processes alter the spatial proximity between cells, further affecting the overall structure of the AT (Marcelin et al. 2022). These findings highlight the importance of 3D analysis for a more precise understanding of AT remodeling in obesity.

Our method also has significant potential for monitoring the effectiveness of nutritional interventions, such as vitamin D and omega‐3 supplementation, in obesity management (Le Jan et al. 2024) as well as strategies to mitigate AT aging (Wang et al. 2022). Additionally, it may be useful in assessing the impact of pharmacological treatments, particularly GLP‐1 receptor agonists, in promoting weight loss and metabolic improvements (Singh et al. 2022). Beyond obesity, this approach could be applied to study adipocyte morphological changes in pathological contexts, such as pericardial AT remodeling in myocardial infarction (Horckmans et al. 2018) or adipocyte function alterations associated with anorexia nervosa (Xiao et al. 2020).

In conclusion, the 3D analysis of AT using tissue clearing without a delipidation step, combined with light sheet microscopy for whole‐sample adipocyte characterization, presents a promising approach for comparing adipocyte morphology in obese versus healthy rats. By refining adipocyte imaging and 3D quantification, our method leads the way for more accurate and comprehensive studies on AT dynamics. This advancement not only deepens our understanding of adipocyte‐driven pathophysiology but also provides a powerful tool for developing and evaluating therapeutic strategies targeting metabolic diseases.

## Author Contributions

**Dylan Le Jan**: conceptualization, analysis, methodology, writing, review and editing. **Manar Harb**: methodology, pipeline image analysis, analysis, writing, review and editing. **Mohamed Siliman Misha**: review and editing. **Jean‐Claude Desfontis**: supervision, review and editing. **Yassine Mallem**: project administration, supervision, review and editing. **Laurence Dubreil**: conceptualization, methodology, image analysis, supervision, validation, writing, review and editing.

## Conflicts of Interest

The authors declare no conflicts of interest.
